# Characterization of the Plasmidome Encoding Carbapenemase and Mechanisms for Dissemination of Carbapenem-Resistant *Enterobacteriaceae*

**DOI:** 10.1128/mSystems.00759-20

**Published:** 2020-11-10

**Authors:** Ryuichiro Abe, Yukihiro Akeda, Yo Sugawara, Dan Takeuchi, Yuki Matsumoto, Daisuke Motooka, Norihisa Yamamoto, Ryuji Kawahara, Kazunori Tomono, Yuji Fujino, Shigeyuki Hamada

**Affiliations:** a Japan-Thailand Research Collaboration Centre on Emerging and Re-emerging Infections, Research Institute for Microbial Diseases, Osaka University, Osaka, Japan; b Department of Anaesthesiology and Intensive Care Medicine, Graduate School of Medicine, Osaka University, Osaka, Japan; c Division of Infection Control and Prevention, Osaka University Hospital, Osaka, Japan; d Division of Infection Control and Prevention, Graduate School of Medicine, Osaka University, Osaka, Japan; e Department of Infection Metagenomics, Research Institute for Microbial Diseases, Osaka University, Osaka, Japan; f Department of Microbiology, Osaka Institute of Public Health, Osaka, Japan; UCSF

**Keywords:** *Enterobacteriaceae*, IMP-1, IMP-6, carbapenem resistance, carbapenemase, chromosomal integration, heteroresistance, plasmid analysis, plasmid dynamics, plasmidome

## Abstract

Carbapenem-resistant *Enterobacteriaceae* (CRE) infections, high in morbidity and mortality, pose serious clinical challenges due to limited treatment options. A previous CRE surveillance study on 1,507 patients from 43 hospitals in Osaka, Japan, revealed that 12% of patients carried CRE and that 95% of the CRE isolates were IMP-type carbapenemase producers. Here, the mechanisms for this regional dissemination of a single carbapenemase gene were investigated. Since the dissemination of CRE is primarily due to the transmission of carbapenemase genes located on plasmids, we analyzed the plasmidome of 230 CRE isolates carrying *bla*_IMP_ by whole-genome sequencing and Southern blotting. *bla*_IMP-6_ was found to be predominantly disseminated among chromosomally distinct isolates through the pKPI-6 plasmid. Underlying the vast clonal dissemination of pKPI-6, various subpopulations deriving from pKPI-6 were identified, which had acquired advantages for the dissemination of CRE isolates. A cluster exhibiting heteroresistance against meropenem by the transcriptional regulation of *bla*_IMP-6_ caused an outbreak likely through covert transmission of *bla*_IMP-6_. For stable carriage of *bla*_IMP-6_, they occasionally integrated *bla*_IMP-6_ on their chromosomes. In addition, we detected one isolate that broadened the range of antimicrobial resistance through a single point mutation in *bla*_IMP-6_ on pKPI-6. Multifaceted analysis of the plasmidome granted us more accurate perspectives on the horizontal spread of CRE isolates, which is difficult to trace only by comparing the whole genomes. This study revealed the predominant spread of a specific carbapenemase-encoding plasmid accompanying the emergence of phenotypically diverse derivatives, which may facilitate further dissemination of CRE in various environments.

**IMPORTANCE** Global dissemination of carbapenem-resistant *Enterobacteriaceae* (CRE) threatens human health by limiting the efficacy of antibiotics even against common bacterial infections. Carbapenem resistance, mainly due to carbapenemase, is generally encoded on plasmids and is spread across bacterial species by conjugation. Most CRE epidemiological studies have analyzed whole genomes or only contigs of CRE isolates. Here, plasmidome analysis on 230 CRE isolates carrying *bla*_IMP_ was performed to shed light into the dissemination of a single carbapenemase gene in Osaka, Japan. The predominant dissemination of *bla*_IMP-6_ by the pKPI-6 plasmid among genetically distinct isolates was revealed, as well as the emergences of pKPI-6 derivatives that acquired advantages for further disseminations. Underlying vast clonal dissemination of a carbapenemase-encoding plasmid, heteroresistance was found in CRE offspring, which was generated by the transcriptional regulation of *bla*_IMP-6_, stabilization of *bla*_IMP-6_ through chromosomal integration, or broadened antimicrobial resistance due to a single point mutation in *bla*_IMP-6_.

## Abstract (precis)

Global dissemination of carbapenem-resistant *Enterobacteriaceae* (CRE) threatens human health by limiting the efficacy of antibiotics even against common bacterial infections. Carbapenem resistance, mainly due to carbapenemase, is generally encoded on plasmids and is spread across bacterial species by conjugation. Most CRE epidemiological studies have analyzed whole genomes or only contigs of CRE isolates. Here, plasmidome analysis on 230 CRE isolates carrying *bla*_IMP_ was performed to shed light into the dissemination of a single carbapenemase gene in Osaka, Japan. The predominant dissemination of *bla*_IMP-6_ by the pKPI-6 plasmid among genetically distinct isolates was revealed, as well as the emergences of pKPI-6 derivatives that acquired advantages for further disseminations. Underlying vast clonal dissemination of a carbapenemase-encoding plasmid, heteroresistance was found in CRE offspring, which was generated by the transcriptional regulation of *bla*_IMP-6_, stabilization of *bla*_IMP-6_ through chromosomal integration, or broadened antimicrobial resistance due to a single point mutation in *bla*_IMP-6_.

## INTRODUCTION

The rapid global dissemination of multidrug-resistant *Enterobacteriaceae* threatens health care systems worldwide (1). Carbapenem-resistant *Enterobacteriaceae* (CRE) are of major concern because alternative treatment options are limited (2). Carbapenem resistance is primarily conferred by carbapenemases that hydrolyze carbapenem (3). KPC, NDM, and OXA-48 are the most commonly detected carbapenemases (3). Carbapenemase genes are generally plasmid encoded and are frequently transmitted across species (4). Therefore, genetic tracking of plasmids encoding carbapenemase genes has allowed the monitoring of the spread of CRE isolates. For example, structural similarities among plasmids from isolates obtained in a single hospital outbreak allowed elucidating links between patients carrying the isolates (5–7), and plasmid data accumulated globally revealed the worldwide spread of an epidemic plasmid carrying *bla*_KPC._(8). However, most regional surveillance studies compared the whole genomes or only contigs of CRE isolates without analyzing the clonality of the spreading carbapenemase-encoding plasmids, and few studies have comprehensively analyzed carbapenemase-encoding plasmids broadly spreading in a certain region (9).

We previously conducted a surveillance study of CRE in 1,507 patients from 43 hospitals in northern Osaka (population, 1,170,000; area, 307 km^2^), Japan (10), and we reported that 12% of the patients carried CRE and 95% of CRE isolates harbored *bla*_IMP-6_, the predominant carbapenemase in Japan. The predominance of this particular carbapenemase gene might have resulted from vigorous horizontal spreading of a specific plasmid carrying *bla*_IMP-6_ in this region. The aim of the present study was to analyze the plasmidome transmitting carbapenemase genes in order to unveil the mechanisms for their regional dissemination.

## RESULTS

### Dissemination of pKPI-6.

**FIG 1:**
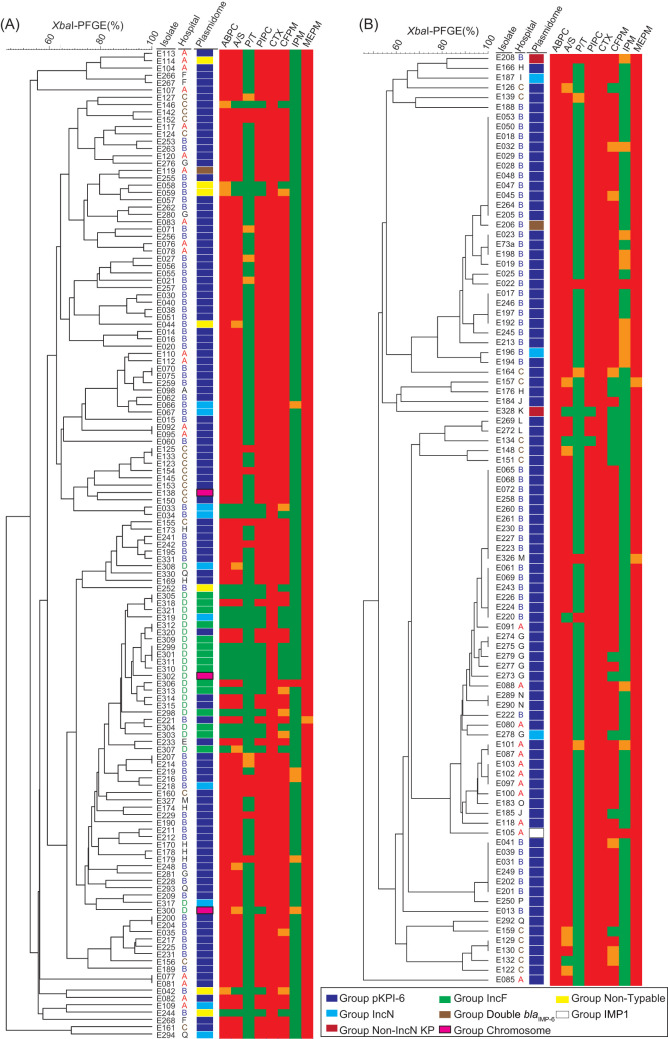
Phylogenetic trees based on XbaI-PFGE and classification of plasmidome carrying *bla*_IMP_ and antimicrobial resistance patterns. The plasmidome carrying *bla*_IMP_ of E. coli (A) and K. pneumoniae (B) isolates was classified according to the size and replicon type of the *bla*_IMP_-carrier plasmids, determined by S1-PFGE and Southern blotting for *bla*_IMP-6_ and *repA* on the IncN plasmid. The plasmidome carrying *bla*_IMP_ was classified as follows: blue, group pKPI-6, pKPI-6-like plasmid (∼50 kbp, encoding *repA* for IncN plasmid); light blue, group IncN, plasmid with *repA* for IncN, but not ∼50 kbp; red, group non-IncN KP, plasmid without *repA* for IncN harbored by K. pneumoniae; green, group IncF, plasmid without *repA* for IncN harbored by E. coli; brown, group double *bla*_IMP-6_, multiple plasmids with *bla*_IMP-6_ carried by a single isolate; enclosed pink, group chromosome, chromosomal *bla*_IMP-6_; yellow, group non-typeable, failure to determine the size of plasmid carrying *bla*_IMP-6_; and white, group IMP1, *bla*_IMP-1_-carrier plasmid. Hospitals where the isolates were obtained are indicated as A to Q. Antimicrobial resistance measured by the broth microdilution method is indicated as follows: red, resistant; orange, intermediate; green, susceptible. Abbreviations: ABPC, ampicillin; A/S, ampicillin/sulbactam; P/T, piperacillin-tazobactam; PIPC, piperacillin; CTX, cefotaxime; CFPM, cefepime; IPM, imipenem; MEPM, meropenem.

All *bla*_IMP_-positive CRE isolates of Escherichia coli (*n* = 135) and Klebsiella pneumoniae (*n* = 95) were classified into seven groups based on the results of S1-PFGE followed by Southern blotting hybridization with probes for the *bla*_IMP_ and *repA* genes encoded on the IncN-type plasmid pKPI-6, sporadically reported as a plasmid carrying *bla*_IMP-6_ (11) (Fig. 1). Ninety-nine of the 135 E. coli isolates (73%) and 88 of the 95 *K. pneumonia* isolates (93%) carried plasmids classified as group pKPI-6 based on plasmid size and replicon type (see Fig. S1 in the supplemental material). Next, we compared the similarity between pKPI-6 and 39 representative plasmids categorized as group pKPI-6 based on whole-genome sequencing (WGS) data using Illumina HiSeq 3000 or Illumina MiSeq (see Fig. S1). The overall sequence identity was 99% ± 0.28%, and the sequence coverage was 98% ± 4.0% (mean ± the standard deviation). The complete sequences of three plasmids were previously confirmed as clonal with pKPI-6 using a combination of PacBio RsII, Illumina HiSeq 3000, and Southern blot methods (12). These analyses confirmed that pKPI-6 was the predominant plasmid responsible for the transmission of *bla*_IMP-6_ in the study area (187 of 230 [81.3%] *bla*_IMP_-positive CRE isolates).

10.1128/mSystems.00759-20.1 FIG S1 Sizes of the plasmids carrying *bla*_IMP_ determined by S1-PFGE, followed by Southern blotting. Phylogenetic trees of E. coli isolates (A) and K. pneumoniae isolates (B) based on XbaI-PFGE and the classification of plasmidome, as indicated in the legend of Fig. 1, are shown. To confirm the similarity of the plasmids in group pKPI-6, the isolates indicated by blue and green arrows were sequenced by Illumina HiSeq 3000 and Illumina MiSeq sequencing, respectively. Download FIG S1, EPS file, 2.6 MB. Copyright © 2020 Abe et al. 2020 Abe et al. This content is distributed under the terms of the Creative Commons Attribution 4.0 International license.

### Genomic analysis of derivatives of the predominant plasmid, pKPI-6.

During the characterization of the *bla*_IMP-6_ plasmids mentioned above, nine E. coli isolates and three K. pneumoniae isolates possessed *bla*_IMP-6_ plasmids categorized as group IncN (Fig. 1). Group IncN *bla*_IMP-6_ plasmids were characterized by replicon type IncN and ranged from 35 to 264 kbp in size, which was different from the pKPI-6 plasmid of 50 kbp (see Fig. S1). The complete sequences of these plasmids indicated that they had preserved the nearly complete locus of pKPI-6 and typically were multireplicon plasmids that had integrated IncF-type plasmids framed by insertion sequences (see Fig. S2A to G and Table S1). In addition, two isolates (E208 and E328) of K. pneumoniae harbored plasmids categorized as group non-IncN KP (Fig. 1B). These plasmids comprised a cassette carrying *bla*_IMP-6_ without IncN-type *repA* of the pKPI-6 plasmid integrated into another plasmid (see Fig. S2H). Interestingly, E. coli isolate E119 and K. pneumoniae isolate E206 coharbored two distinct *bla*_IMP-6_-encoding plasmids of different sizes and were categorized as group double *bla*_IMP-6_ (Fig. 1; see also Fig. S3A). Barring occasional isolations of organisms coharboring different carbapenemase genes (13, 14), few studies have shown the coexistence of two identical carbapenemase genes on different plasmids within an isolate (15). WGS revealed that isolate E119 carried pKPI-6 and an IncF-type plasmid (pEC743_1) that had a *bla*_IMP-6_ cassette from pKPI-6 integrated (49) (see Fig. S3B and C).

**FIG 2:**
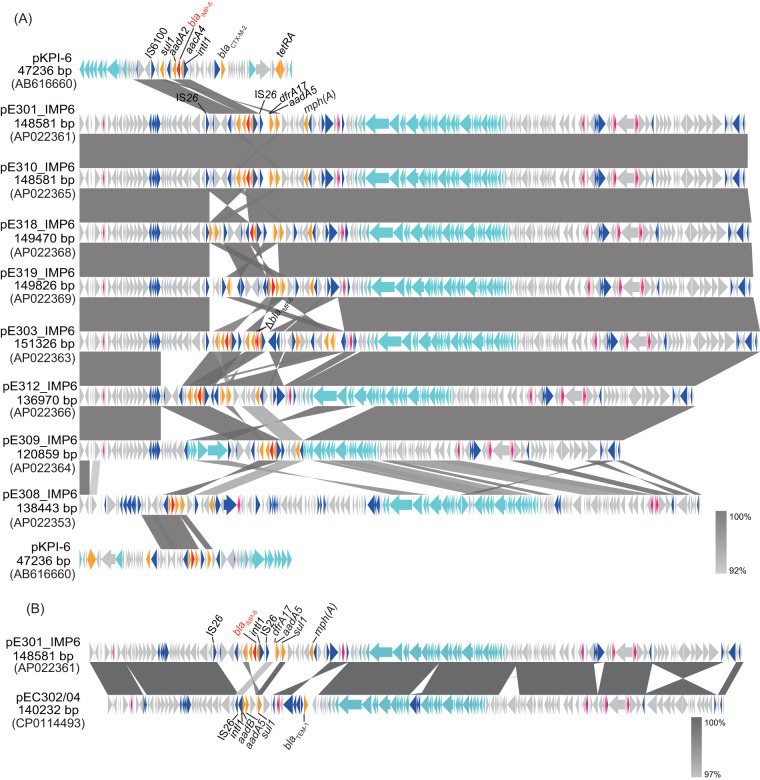
Comparison of plasmids in group IncF and the ancestor of these plasmids. (A) Comparison of plasmids in group IncF with plasmid pKPI-6. In addition to showing high similarity to each other, the region containing *bla*_IMP-6_ bracketed by a set of IS*26* was identical to a part of pKPI-6. Block arrows indicate confirmed or putative open reading frames (ORFs), and their orientations. Arrow size is proportional to the predicted ORF length. The color code is as follows: red, carbapenem resistance gene; yellow, other antimicrobial resistance gene; light blue, conjugative transfer gene; blue, mobile element; and purple, toxin-antitoxin. Putative, hypothetical, or unknown genes are represented as gray arrows. The gray-shaded area indicates regions with high identity between the two sequences. Accession numbers of the plasmids are indicated in brackets. (B) Ancestor of plasmid pE301_IMP6. The backbone of plasmid pE301_IMP6 which is representative of the plasmids in group IncF, corresponded to the structure of plasmid pEC302_04 reported in Malaysia in 2004.

10.1128/mSystems.00759-20.2 FIG S2 Comparisons of plasmids in group IncN and group Non-IncN with pKPI-6 plasmid. (A) Comparison of plasmids pKPI-6 and pE034_IMP6 (group IncN). From the similarity between the plasmids, pE034_IMP6 was assumed to have incorporated pKPI-6 plasmid. During integration, a set of IS*Sbo1* bracketed pKPI-6, breaking *bla*_CTX-M-2_, resulting in target sight duplication (TSD) shown beside each IS*Sbo1*. The color code is the same as that described in the legend of Fig. 2. (B) Similarity of pE034_IMP6 and pE033_IMP6 (group IncN). pE033_IMP6 was identical to pE034_IMP6 except for a short region inserted between a set of IS*Sbo1*. *bla*_CTX-M-2_ encoded on both plasmids was broken in the process of incorporation of the pKPI-6. (C) Comparison of plasmids pKPI-6, pE278_IMP6, and pE196_IMP6 (group IncN). pE278_IMP6 was assumed to have incorporated the pKPI-6 plasmid, bracketed by a set of IS*26*. *repA* on pE278_IMP6 was divided into two regions by insertion of IS*26*. pE196_IMP6 had a structure similar to that of pE278_IMP6. The mechanism of integration of pKPI-6 into pE196_IMP6 was previously reported (12). (D) Comparison of plasmids pKPI-6 and pE294_IMP6 (group IncN). pE294_IMP6 seemed to have incorporated pKPI-6 plasmid, bracketed by a set of IS*15*. (E) Comparison of plasmids pKPI-6 and pE317_IMP6 (group IncN). pE317_IMP6 was assumed to have incorporated the pKPI-6 plasmid, bracketed by a set of IS*26*. IS*6100* on pKPI-6 was divided into two regions during this integration. (F) Comparison of plasmids pKPI-6 and pE109_IMP6 (group IncN). In plasmid pE109_IMP6, a conjugative transfer region was deleted from plasmid pKPI-6. We speculate that this is why plasmid pE109_IMP6 was not self-transmissible. (G) Comparison of plasmids pE308_IMP6 and pE319_IMP6 (group IncN). Plasmids pE308_IMP6 and pE319_IMP6 were categorized as group IncN according to Southern hybridization analysis because *repA* or Δ*repA* was embedded in plasmids pE308_IMP6 and pE319_IMP6, respectively. Apart from other plasmids in group IncN, these plasmids had incorporated only a part of pKPI-6. The structures of these plasmids were similar to those of plasmids in group IncF (Fig. 2A). (H) Comparison of plasmids pE328_IMP6 and pE208_IMP6 (group non-IncN KP) with plasmid pKPI-6. Apart from the plasmids in group IncN, these plasmids had lost *bla*_CTX-M-2_ and incorporated only a part of pKPI-6, including *bla*_IMP-6_. IS*26* next to the region embedding a part of pKPI-6 seemed to work in each integration. Download FIG S2, EPS file, 2.7 MB. Copyright © 2020 Abe et al. 2020 Abe et al. This content is distributed under the terms of the Creative Commons Attribution 4.0 International license.

10.1128/mSystems.00759-20.3 FIG S3 Comparison of the plasmids carried by E. coli isolate E119 with plasmid pKPI-6 and the putative ancestor of plasmid pE119_6kIMP6. (A) Comparisons of the sizes of plasmids for pE119_5kIMP6 and pE119_6kIMP6 (group double *bla*_IMP-6_) to that of pE188_IMP6 (group pKPI-6). PFGE of S1-digested genomic DNA from E. coli isolate E119 and K. pneumoniae isolate E188, followed by Southern blotting with a *bla*_IMP-6_ probe, indicated the presence of both plasmids pE119_5kIMP6 and pE119_6kIMP6 in E. coli isolate E119. M, DNA size marker (lambda ladder; Bio-Rad). Arrows indicate chromosome band, plasmid pE119_5kIMP6 (50 kbp), plasmid pE119_6kIMP6 (60 kbp), and plasmid pE188_IMP6 (50 kbp). (B) Comparison of plasmids pE119_5kIMP6 and pE119_6kIMP6 to plasmid pKPI-6. Isolate E119 possessed both plasmids pE119_5kIMP6 and pE119_6kIMP6 and was categorized as group double *bla*_IMP-6_. pE119_5kIMP6 showed high similarity with pKPI-6. Meanwhile, in pE119_6kIMP6, only a 14-kbp region containing *bla*_IMP-6_ juxtaposed with a set of IS*15* showed high similarity with plasmid pKPI-6, and it did not carry *bla*_CTX-M-2_ and *repA* for IncN plasmid. (C) Ancestor of plasmid pE119_6kIMP6. pE119_IMP6 consisted of an insertion of a 14-kbp region in plasmid pKPI-6 and an ∼50-kbp region that seemed to have originated from pEC743_1 (CP015070) as reported in Dubai, United Arab Emirates in 2012 (Antimicrob Agents Chemother 60:6948–6951, 2016, http://doi:10.1128/AAC.01130-16). The color code is the same as that described in the legend of Fig. 2. Download FIG S3, EPS file, 2.6 MB. Copyright © 2020 Abe et al. 2020 Abe et al. This content is distributed under the terms of the Creative Commons Attribution 4.0 International license.

10.1128/mSystems.00759-20.6 TABLE S1 Replicon types of *bla*_IMP-6_-carrier plasmids from representative isolates in each *bla*_IMP_ carriage group. The complete sequences of plasmids carrying *bla*_IMP-6_ were determined by Nanopore GridION or PacBio RSII combined with Illumina MiSeq or Illumina HiSeq 3000 sequencing. Replicon types of plasmids were analyzed using PlasmidFinder. Plasmid groups of the isolates are indicated in Fig. 1. Download Table S1, PDF file, 0.3 MB. Copyright © 2020 Abe et al. 2020 Abe et al. This content is distributed under the terms of the Creative Commons Attribution 4.0 International license.

### Characterization of IncF plasmids encoding *bla*_IMP-6_.

In addition to the K. pneumoniae isolates carrying group non-IncN KP plasmids, E. coli isolates carrying plasmids without IncN replicon were found in a single hospital (hospital D; Fig. 1A). WGS of these isolates revealed that they harbored nearly identical *bla*_IMP-6_-encoding plasmids with an IncFIA-type replicon (categorized as group IncF) (Fig. 2A; see also Table S1). These plasmids were generated by integration of a cassette carrying *bla*_IMP-6_ on pKPI-6 into another IncF plasmid at IS*26*. This IncF plasmid (pEC302/04; Fig. 2B) has been reported to transmit antimicrobial resistance since 1965 (16).

The MICs of meropenem for the E. coli isolates carrying group IncF plasmids were low compared to those of E. coli isolates harboring other *bla*_IMP-6_-encoding plasmids, such as pKPI-6 (see Fig. S4). Mutations or deletions in the porin (OmpF) gene in E. coli have been reported to enhance resistance to β-lactams (17). However, all E. coli isolates carrying group IncF plasmids had a premature termination codon within *ompF*, whereas the other isolates carried wild-type *ompF* (Table 1; see also Table S2). MICs of meropenem were low for these group IncF plasmid-carrying isolates, despite them being OmpF deficient. To investigate carbapenem resistance in the same genetic background, plasmids from representative isolates in each *bla*_IMP-6_ carriage group were transformed into the E. coli TOP10 strain and MICs for the transformants were determined. Transformant T305 carrying pE305_IMP6_single_ of group IncF from E. coli isolate E305 was more susceptible to meropenem than transformants carrying *bla*_IMP-6_-harboring plasmids of groups (Table 2). The transcription of *bla*_IMP-6_ in the pE305_IMP6_single_ transformant was significantly lower than that in the pKPI-6 transformant (see Fig. S5A), although the plasmid copy numbers in the bacterial cells were comparable (see Fig. S5B). These results indicated that the lower MICs of meropenem in E. coli isolates carrying group IncF plasmids were due to the reduced transcription of *bla*_IMP-6_.

**TABLE 1:** Numbers of isolates carrying a porin gene with mutation(s)^*a*^

Strain	Group	No. of isolates carrying mutation(s)			Total no. of sequenced isolates
		*ompC ompK35*	*ompF ompK36*	*ompC*+*ompF ompK35+ompK36*	
E. coli	pKPI-6	0	2	0	14
	IncN	1	2	1	9
	IncF	0	11	0	11
	Double *bla*_IMP_	0	0	0	1
	Chromosome	0	2	0	3
					
K. pneumoniae	pKPI-6	1	7	0	29
	IncN	0	1	0	2
	Non-IncN KP	0	0	0	2

a Groups correspond to those in Fig. 1. E. coli and K. pneumoniae isolates were sequenced using Illumina HiSeq 3000 or Illumina MiSeq, and the sequences were compared to the following reference sequences: *ompC* and *ompF* sequences for E. coli strain MG1655 (K-12 substrain) and *ompK35* and *ompK36* sequences for K. pneumoniae strain ATCC 13883. Mutant porin was defined as having <90% identity or <90% coverage.

**TABLE 2:** MICs of meropenem and conjugation efficiency in transformants with plasmids from representative isolates in each group^*a*^

Original species	Group	Original host isolate	MIC (mg/liter)	Avg conjugation efficiency ± SD
E. coli	pKPI-6	E174	4	(8.1 ± 3.8) × 10^−2^
	IncN	E066	16	(2.2 ± 3.1) × 10^−4^
	IncN	E033	16	(2.4 ± 1.3) × 10^−2^
	IncF	E305	<1	(7.5 ± 2.3) × 10^−4^
				
K. pneumoniae	pKPI-6	E188	4	(3.7 ± 2.0) × 10^−1^
	IncN	E187	4	(2.9 ± 1.1) × 10^−1^
	IncN	E196	16	(4.4 ± 3.5) × 10^−1^
	Non-IncN KP	E208	4	0
	Non-IncN KP	E328	2	(3.1 ± 2.6) × 10^−4^

a Groups correspond to those in Fig. 1. Plasmids from representative isolates in each group were transformed into E. coli TOP10 strain by electroporation. MICs of meropenem for these transformants were measured by the broth microdilution method, in triplicate. The conjugation assay was conducted by mating the transformants as donors and E. coli TUM3456 as a recipient. The conjugation frequency was calculated as the CFU number of transconjugants per number of donors plus transconjugants. Average conjugation efficiencies from triplicate assays are indicated.

10.1128/mSystems.00759-20.4 FIG S4 MICs of meropenem for CRE isolates. MICs of meropenem for E. coli (A) and K. pneumoniae (B) isolates are shown as bar graphs. MICs were measured using ETEST. MICs higher than 32 μg/ml are reported as 32 μg/ml. The phylogenetic tree of isolates based on XbaI-PFGE and the classification of plasmidome are the same as in Fig. 1. Download FIG S4, PDF file, 0.6 MB. Copyright © 2020 Abe et al. 2020 Abe et al. This content is distributed under the terms of the Creative Commons Attribution 4.0 International license.

10.1128/mSystems.00759-20.5 FIG S5 Transcription of *bla*_IMP-6_ in E. coli isolate E305. (A) Transcript levels of *bla*_IMP-6_ from plasmids pE305_IMP6_single_ (group IncF) and pE188_IMP6 (group pKPI-6). qPCR demonstrated significantly lower transcription of *bla*_IMP-6_ in pE305_IMP6_single_-transformant E. coli T305 cells than in pE188_IMP6-transformant E. coli T188 cells (*P* = 0.0055). The bar chart represents the relative mRNA transcript ratio of *bla*_IMP-6_ to the housekeeping gene, *rrsA*, which was used as a reference gene. Bars indicate means ± standard deviations calculated from sextuplet experiments. The *P* value was calculated by using the Mann-Whitney U test. (B) Cellular copy numbers of plasmids pE305_IMP6_single_ and pE188_IMP6. qPCR demonstrated that the copy numbers of pE188_IMP6 in strain T188 and pE305_IMP6_single_ in strain T305 were not significantly different. The bar chart represents the DNA copy number ratio of *bla*_IMP-6_ in strain T188 or *repA2* on plasmid in strain T305 to chromosomal *rrsA*, which was used as an internal control. Bars indicate means ± standard deviations calculated from sextuplet experiments. (C) Copy numbers of *bla*_IMP-6_ on plasmids pE305_IMP6 and pE318_IMP6 (group IncF). qPCR demonstrated that pE305_IMP6 carried three copies of *bla*_IMP-6_, whereas pE318_IMP6 carried one copy. The bar chart represents the DNA copy number ratio of *bla*_IMP-6_ to *repA2* on IncF plasmid, used as an internal control gene. Bars indicate means ± standard deviations, calculated from quintuplicate experiments. (D) Cellular copy numbers of plasmids pE305_IMP6 and pE318_IMP6. qPCR demonstrated that the copy numbers of pE305_IMP6 in isolate E305 and pE318_IMP6 in isolate E318 were not significantly different. The bar chart represents the DNA copy number ratio of *repA2* to chromosomal *rrsA*, which was used as an internal control. Bars indicate means ± standard deviations, calculated from sextuplet experiments. (E) Sizes of plasmids pE305_IMP6 and pE305_IMP6_single_. PFGE of S1-digested genomic DNA from E. coli isolates E305 and T305, followed by Southern blotting with a *bla*_IMP-6_ probe indicated the size of each plasmid. M, DNA size marker (lambda ladder; Bio-Rad). (F) Copy numbers of *bla*_IMP-6_ on plasmid pE305_IMP6 and plasmid pE305_IMP6_single_ in transformant E. coli strain T305. Plasmid pE305_IMP6 carried three copies of *bla*_IMP-6_, whereas plasmid from transformant T305 carried one copy. Bars indicate means ± standard deviations, calculated from quintuplicate experiments. Download FIG S5, EPS file, 2.8 MB. Copyright © 2020 Abe et al. 2020 Abe et al. This content is distributed under the terms of the Creative Commons Attribution 4.0 International license.

10.1128/mSystems.00759-20.7 TABLE S2 (A) Comparison of *ompC* and *ompF* harbored by E. coli isolates evaluated and an E. coli reference strain. Groups correspond to those in Fig. 1. E. coli isolates were sequenced using Illumina HiSeq 3000 or Illumina MiSeq. Protein indicates the length of the amino acids encoded on the *ompC* or *ompF* sequence in E. coli strain MG1655 (K-12 substrain). Query lengths of amino acids = 367 for OmpC and 362 for OmpF. A comparison with the sequence of an E. coli reference strain is shown (%). PSC, premature stop codon. (B) Comparison of *ompK35* and *ompK36* harbored by the *K. pneumoniae* isolates evaluated and a K. pneumoniae reference strain. Groups correspond to those in Fig. 1. K. pneumoniae isolates were sequenced using Illumina HiSeq 3000 or Illumina MiSeq. Protein indicates the length of the amino acids encoded on the *ompK35* or *ompK36* sequence in K. pneumoniae strain ATCC 13883. Query lengths of amino acids = 359 for *ompK35* and 372 for *ompK36*. Comparison with the sequence of a K. pneumoniae reference strain is shown. The loop3 structure in *ompK36* was preserved in all isolates, except isolate E139. Download Table S2, PDF file, 0.7 MB. Copyright © 2020 Abe et al. 2020 Abe et al. This content is distributed under the terms of the Creative Commons Attribution 4.0 International license.

### Heteroresistance to carbapenems: enhanced resistance through gene amplification.

E. coli isolates E305 and E318 were found to carry group IncF plasmids, and WGS revealed that their chromosomes were nearly identical (query: E318, identity 100%, coverage 100%; query: E305, identity 100%, coverage 98% [in BLASTN]). Isolate E318 harbored genes encoding extended-spectrum β-lactamases (ESBLs), such as *bla*_CTX-M-14_ and *bla*_TEM-1B_, on a plasmid other than pE318_IMP6, whereas isolate E305 did not have these genes (Table 3). IMP-6 confers resistance to cephalosporins and meropenem but hydrolyzes penicillins very poorly (18). Therefore, isolate E318 exhibited broader antimicrobial resistance than isolate E305. In contrast, the MIC of meropenem for E305 was higher than that for E318.

**TABLE 3:** Comparison of E. coli isolates E305 and E318^*a*^

*E. coli* isolate	Group	Antimicrobial MIC (mg/liter)								Porin		Meropenem MIC (mg/liter)		ESBL	
		ABPC	A/S	P/T	PIPC	CTX	CFPM	IPM	MEPM	OmpC	OmpF	Etest	BMD	Plasmid	Others
E305	IncF	≤8 (S)	≤8/4 (S)	≤16 (S)	≤8 (S)	>2 (R)	>16 (R)	≤1 (S)	>2 (R)	W/T	PSC	>32	16	(–)	(–)
E318	IncF	>16 (R)	>16/8 (R)	≤16 (S)	>64 (R)	>2 (R)	>16 (R)	≤1 (S)	>2 (R)	W/T	PSC	4	8	(–)	*bla*_CTX-M-14_, *bla*_TEM-1B_

a Groups correspond to those presented in Fig. 1. ABPC, ampicillin; A/S, ampicillin/sulbactam; P/T, piperacillin-tazobactam; PIPC, piperacillin; CTX, cefotaxime; CFPM, cefepime; IPM, imipenem; MEPM, meropenem. R or S in parentheses indicates resistance or susceptibility, respectively, based on CLSI M200-S26. W/T, wild type; PSC, premature stop codon. Meropenem MICs were measured using either the Etest or broth microdilution (BMD). ESBL genes encoded on *bla*_IMP-6_-carrier plasmid (Plasmid) and on others (Others) are indicated in the last two columns.

**FIG 3:**
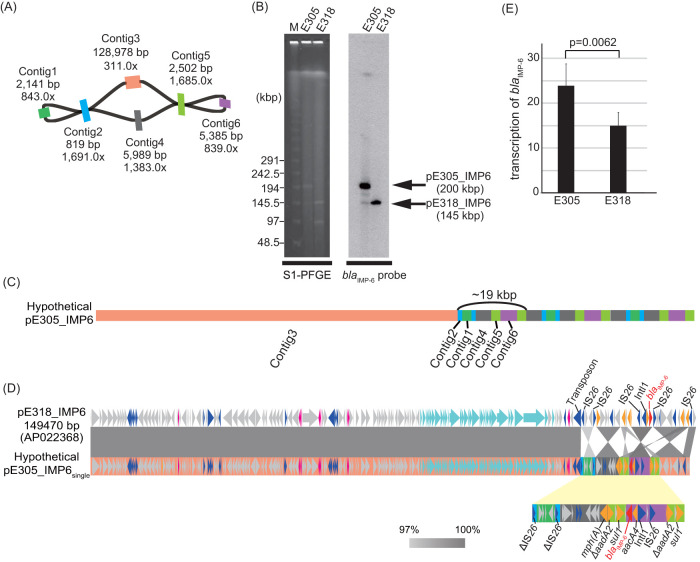
Genomic structure of group IncF plasmid pE305_IMP6 and enhanced transcription of *bla*_IMP-6_. (A) Genomic structure of plasmid pE305_IMP6. *De novo* assembly graph of plasmid pE305_IMP6 visualized by Bandage displays the connections between contigs. The length and depth of each contig are shown. Contig2 connects Contig1 with Contig3 or Contig4, and Contig5 connects Contig6 with Contig3 or Contig4. (B) Sizes of plasmids pE305_IMP6 and pE318_IMP6. PFGE of S1-digested genomic DNA from E. coli isolates E305 and E318, followed by Southern blotting with a *bla*_IMP-6_ probe, indicated the size of each plasmid. M, DNA size marker (lambda ladder; Bio-Rad). (C) Hypothetical structure of pE305_IMP6. The colors correspond to the colors of contigs in panel A. (D) Genomic comparison of pE318_IMP6 and hypothetical pE305_IMP6_single_. According to the overlap between contigs of pE305_IMP6, the hypothetical sequence shown was assembled and compared to the sequence of plasmid pE318_IMP6. Except for the repeats, pE305_IMP6_single_ and pE318_IMP6 were highly similar. Block arrows indicate confirmed or putative ORFs and their orientations. The color code, arrows, and similarity are as described in the legend of Fig. 2. The colors under arrows of pE305_IMP6_single_ correspond to the colors of contigs in panel A. (E) Transcript levels of *bla*_IMP-6_ in E. coli isolates E305 and E318. qPCR revealed significantly higher transcription of *bla*_IMP-6_ in isolate E305 than in isolate E318. The bar chart represents the mRNA transcript ratio of *bla*_IMP-6_ to the housekeeping gene *rrsA*, which was used as a reference gene. Bars indicate means ± standard deviations, calculated from sextuplet experiments. The *P* value was calculated by using the Mann-Whitney U test.

WGS of E305 and E318 revealed the complete sequence of pE318_IMP6; however, it failed to determine the complete sequence of pE305_IMP6. Therefore, to analyze the structure of pE305_IMP6, we used a combination of WGS, Southern blotting, and qPCR analysis. The length and depth of each contig of pE305_IMP6 deduced from WGS are shown in the *de novo* assembly graphs generated using the Bandage software (19) in Fig. 3A. The total length of pE305_IMP6 deduced from WGS data were ∼149 kbp. However, according to Southern blotting results, pE318_IMP6 and pE305_IMP6 were ∼145 and ∼200 kbp in size, respectively (Fig. 3B). Based on the depth of each contig, the copy number of each contig was predicted as follows: Contig3, 1 copy; Contig2 and Contig5, 6 copies; Contig1 and Contig6, 3 copies; and Contig4, 5 copies (Fig. 3A). Therefore, pE305_IMP6 was predicted to have an ∼19-kbp repeat region consisting of triplication of Contig1 and Contig6, sextuplication of Contig2 and Contig5, and quintuplication of Contig4 (Fig. 3C). Except for the repeat region, pE305_IMP6 and pE318_IMP6 exhibited high sequence similarity (identity, 99.27%; coverage, 100%) (Fig. 3D). The *bla*_IMP-6_ gene was located on Contig6 and was predicted to be triplicated. qPCR analysis corroborated that pE305_IMP6 carried three copies of *bla*_IMP-6_, whereas pE318_IMP6 harbored a single copy (see Fig. S5C). *bla*_IMP-6_ transcription was significantly higher in isolate E305 than in isolate E318 (Fig. 3E), even though the *bla*_IMP-6_-carrier plasmid copy numbers in the cells of these isolates were not significantly different (see Fig. S5D). Triplication of *bla*_IMP-6_ in tandem resulted in a higher transcription level in E305 and thus a higher level of resistance to meropenem.

Subculture of the clonal isolate E305 in broth medium revealed a mixture of subpopulations of bacteria carrying a plasmid with multiple *bla*_IMP-6_ copies (which represented the majority) and bacteria carrying a plasmid with a single *bla*_IMP-6_ copy. In Southern blotting analyses for *bla*_IMP-6_, a faint band at ∼145 kbp was observed in addition to the major band at ∼200 kbp (Fig. 3B). It was also found that T305 (a transformant of pE305_IMP6_single_ extracted from E305) carried an ∼145-kbp plasmid without *bla*_IMP-6_ amplification due to *recA* deficiency in the recipient E. coli TOP10 strain (see Fig. S5E) (20). qPCR analysis confirmed that T305 carried one *bla*_IMP-6_ copy on its plasmid (see Fig. S5F). These results indicated the existence of a subpopulation carrying a plasmid with one *bla*_IMP-6_ copy within E. coli isolate E305, whereas the majority of the population carried a plasmid harboring three copies of *bla*_IMP-6_.

### Comparison of CRE isolates carrying pKPI-6 with those carrying other groups of plasmids harboring *bla*_IMP-6_.

*bla*_CTX-M-2_, which is an ESBL gene located distant from *bla*_IMP-6_ on pKPI-6, compensated for the narrow range of hydrolysis of β-lactams by IMP-6 (11, 18). However, these two β-lactamase genes were not always transferred together from pKPI-6 to another plasmid. Plasmids categorized as group non-IncN KP and group IncF did not carry ESBL genes (see Table S3) and rarely conferred resistance to penicillins, in contrast to pKPI-6, which confers broad resistance to β-lactams (Fig. 1). We next measured the conjugation efficiency of representative plasmids in each group (Table 2). pKPI-6 plasmids and group IncN plasmids, which had the entire pKPI-6 plasmid incorporated, showed a higher conjugation efficiency than group non-IncN KP/IncF plasmids. These characteristics may have facilitated the vast horizontal dissemination of pKPI-6 in the study area.

10.1128/mSystems.00759-20.8 TABLE S3 Location of ESBL genes carried *bla*_IMP_-carrier isolates. Groups correspond to those in Fig. 1. The complete sequences of *bla*_IMP-6_-carrier plasmids were determined by Nanopore GridION sequencing combined with Illumina MiSeq or Illumina HiSeq 3000 sequencing. ESBL genes were detected using ResFinder. Download Table S3, PDF file, 0.5 MB. Copyright © 2020 Abe et al. 2020 Abe et al. This content is distributed under the terms of the Creative Commons Attribution 4.0 International license.

Compared with the chromosomal diversity among E. coli isolates bearing pKPI-6, K. pneumoniae isolates carrying pKPI-6 exhibited higher clonality as indicated by pulsed-field gel electrophoresis with XbaI (XbaI-PFGE) analysis (Fig. 1). This may be explained by the presence of the *kikA* gene on pKPI-6, the product of which reportedly promotes cell death of K. pneumoniae following conjugation (21). The conjugation efficiency of pKPI-6 into K. pneumoniae ATCC 13883 was considerably lower than that into E. coli TUM3456 (3.3 × 10^−4^ and 3.7 × 10^−1^, respectively). Maybe only “*kikA*-resistant” K. pneumoniae are able to acquire pKPI-6, leading to clonal similarity among the K. pneumoniae isolates bearing pKPI-6.

### Chromosomal integration of *bla*_IMP-6_.

**FIG 4:**
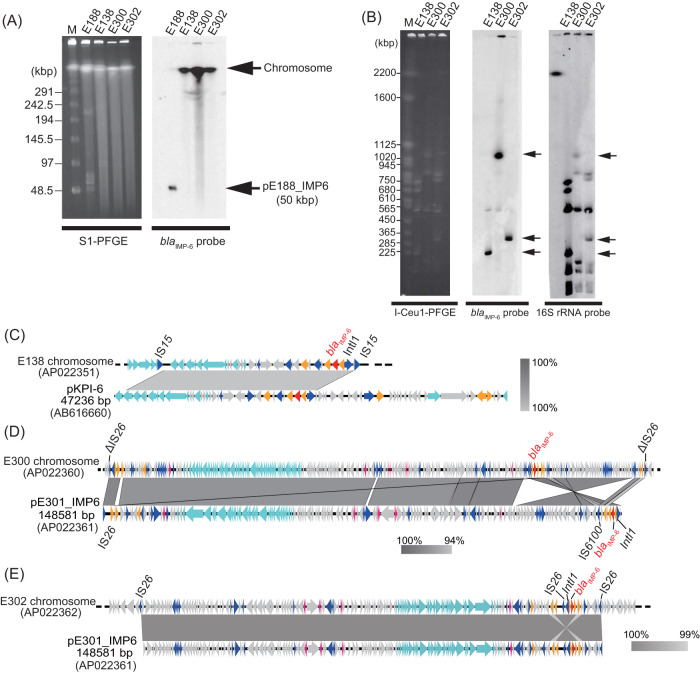
Chromosomal integration of *bla*_IMP-6_. (A) S1-PFGE followed by Southern blotting with a *bla*_IMP-6_ probe. Arrows indicate the segments carrying *bla*_IMP-6_ in E. coli isolates E138, E300, and E302 (group chromosome) and K. pneumoniae isolate E188 (group pKPI-6). M, DNA size marker (lambda ladder; Bio-Rad). (B) I-Ceu1-PFGE followed by Southern blotting with *bla*_IMP-6_ and 16S rRNA probes. Arrows indicate the segments encoding *bla*_IMP-6_ or 16S rRNA, proving that *bla*_IMP-6_ and 16S rRNA were located on the same segment. M, DNA size marker (Saccharomyces cerevisiae ladder; Bio-Rad). (C) Chromosomal integration of a region carrying *bla*_IMP-6_ in isolate E138. A 23-kbp region containing *bla*_IMP-6_ of plasmid pKPI-6 was integrated in the chromosome of isolate E138. This region was bracketed by a set of IS*15*. (D) Comparison of the chromosomal genomic structure of isolate E300 with plasmid pE301_IMP6. Isolate E300 carried chromosomal *bla*_IMP-6_, and the region bracketed by a set of mutated IS*26* showed high similarity with plasmid pE301_IMP6 in group IncF. (E) Chromosomal integration of plasmid pE301_IMP6 in isolate E302. Isolate E302 acquired chromosomal *bla*_IMP-6_ by incorporation of plasmid pE301_IMP6 bracketed by a set of IS*26*. The color code is the same as that described in the legend of Fig. 2.

Unlike most CRE isolates, which carried the predominant pKPI-6 or other *bla*_IMP-6_-encoding plasmids, 3 of 135 E. coli isolates (E138, E300, and E302) harbored *bla*_IMP-6_ on their chromosomes, as indicated by S1-PFGE followed by Southern blotting with *bla*_IMP-6_ probes (Fig. 1A and Fig. 4A). I-CeuI-PFGE followed by Southern blotting with probes for the *bla*_IMP-6_ and 16S rRNA genes confirmed chromosomally located *bla*_IMP-6_ (Fig. 4B). WGS revealed that the chromosome of isolate E138 had a cassette harboring *bla*_IMP-6_ integrated, framed by a set of IS*15* (Fig. 4C). The chromosomes of E300 and E302 had IncFIA plasmids carrying *bla*_IMP-6_ integrated (Fig. 4D and E). Although these plasmids were essentially identical to pE301_IMP6 (E. coli, group IncF), these isolates were phylogenetically distinct on the XbaI-PFGE phylogenetic tree (Fig. 1).

### Emergence of pKPI-6-like plasmid harboring *bla*_IMP-1_.

**FIG 5:**
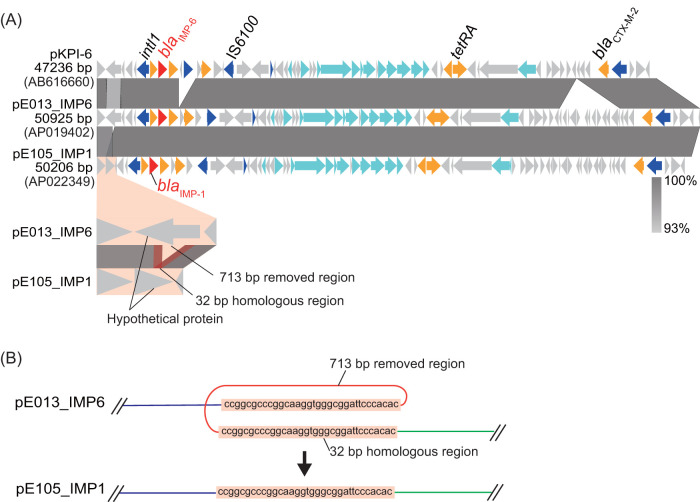
Plasmid pE105_IMP1 carrying *bla*_IMP-1_ was derived from plasmid pKPI-6 by homologous recombination. (A) Comparison of the pE105_IMP1 and pKPI-6 plasmids. The genomic structure of pE105_IMP1 (group IMP1) was compared to plasmids pKPI-6 and pE013_IMP6 (group pKPI-6) obtained from K. pneumoniae isolate E013. Differences between pE105_IMP1 and pE013_IMP6 are visually extended at the bottom. The color code is the same as that described in the legend of Fig. 2. (B) Schematic chart of homologous recombination. The 713-bp region of plasmid pE013_IMP6 was removed by homologous recombination at the 32-bp region.

One K. pneumoniae isolate, E105, harbored *bla*_IMP-1_, which is a single-nucleotide variant of *bla*_IMP-6_, within a clonal cluster of pKPI-6 carriers (Fig. 1B). Due to this mutation, E105 was resistant to imipenem, whereas most isolates carrying *bla*_IMP-6_ were susceptible to this antibiotic. WGS revealed that plasmids pKPI-6, pE013_IMP6 (plasmid group pKPI-6), and pE105_IMP1 were 99.8% identical, with a coverage of 100% (query: pE013_IMP6) (Fig. 5). The only difference was the presence of a 714-bp region bracketed by a set of homologous regions in pE013_IMP6.

## DISCUSSION

IMP-producing *Enterobacteriaceae* have been reported sporadically on a global basis (2). IMP-4-producing *Enterobacteriaceae* are endemic to Australia (22), and IMP-1, -4, and -8 producers have been occasionally detected in China (23). Our study revealed the exclusive dissemination of IMP-6 producers (95% of CRE isolates) in northern Osaka, Japan, consistent with findings in previous studies (11, 24, 25). By analyzing the plasmidome transmitting *bla*_IMP_, we clarified the relationships between *bla*_IMP_-harboring isolates that seemed diverse based on XbaI-PFGE analysis or comparison of short-read WGS results.

The present study revealed predominant dissemination of pKPI-6 in the study area, which may have resulted in the emergence of diverse derivatives. Group IncF plasmids possessed similar genomic structures, consisting of the globally disseminated IncF plasmid and a *bla*_IMP-6_ cassette cointegrated on the pKPI-6 genome, without accompaniment of *bla*_CTX-M-2_ (Fig. 2). Our analysis revealed that *bla*_IMP-6_ transcription was lower from group IncF plasmid (pE305_IMP6_single_) than from pKPI-6 in E. coli cells of the same genetic background (see Fig. S5A). Low carbapenemase gene transcription is considered one of the reasons for reduced resistance to meropenem (26). Therefore, CRE isolates carrying group IncF plasmids might have a reduced fitness cost for the carriage of *bla*_IMP-6_, leading to further environmental dissemination of *bla*_IMP-6_ (27).

Unlike for other plasmids in group IncF, the complete sequence of pE305_IMP6 could not be obtained by long-read or short-read sequencing because of a signature 19-kbp repeat sequence unit. Based on combined WGS, Southern blotting, and qPCR data, we proposed a hypothetical structure of pE305_IMP-6 (Fig. 3C). Our results indicated that, despite its clonal origin, CRE isolate E305 comprised two different populations: a major population carrying pE305_IMP-6 with multiple *bla*_IMP-6_ copies and a minor population carrying pE305_IMP-6_single_ with a single *bla*_IMP-6_ copy (Fig. 3B; see also Fig. S5E and F). Moreover, the amplification of *bla*_IMP-6_ on the IncF plasmid enhanced the transcription of *bla*_IMP-6_ (Fig. 3E), resulting in increased resistance to meropenem (Table 3). These results are consistent with previous studies reporting higher resistance to carbapenem through amplification of *bla*_OXA-58_ (28) and *bla*_NDM-1_ (20).

All E. coli isolates carrying group IncF plasmids were found to possess *ompF* with a premature termination codon (see Table S2). When an isolate producing wild-type OmpF carries this plasmid with a single copy of *bla*_IMP-6_, the isolate is difficult to detect due to weaker resistance to meropenem. However, when an isolate with a porin mutation acquires a group IncF plasmid with multiple *bla*_IMP-6_ copies, it may abruptly exhibit strong resistance to meropenem without any direct trace of horizontal transfer. These types of plasmids may act as “hidden transmitters” of *bla*_IMP-6_.

Moreover, we demonstrated chromosomal integration of group IncF plasmids in some E. coli isolates. Carbapenemase genes have been reported to be transmitted primarily through plasmid conjugation (4), and chromosomal integration has been reported in a limited number of strains (29). In our study, 3 of 135 E. coli isolates (2.2%) exhibited chromosomal integration of *bla*_IMP-6_, which presumably occurred during the vast horizontal spread of pKPI-6. Compared to *bla*_IMP-6_ on plasmids, chromosomal *bla*_IMP-6_ was not readily transmissible to another patient. However, these isolates may stably possess *bla*_IMP-6_ within a patient and not lose carbapenem resistance through the elimination of plasmids harboring *bla*_IMP-6_.

In the early 1990s, some unique metallo-β-lactamases were reported in Japan (30, 31), followed by the identification of IMP-1 (32). Since then, these β-lactamases have been frequently identified in Japan (33). The single amino acid variant, IMP-6, was identified in 2001 (18). IMP-1 producers have disseminated mainly in eastern Japan, including Tokyo (24, 34), whereas IMP-6 producers have been almost exclusively found in western Japan, including Osaka (7, 10, 11, 25). Consistent with these findings, in the present study only one K. pneumoniae isolate carrying *bla*_IMP-1_, E105, was isolated in hospital A, where CRE carrying pKPI-6 were dominant. The patient carrying CRE isolate E105 was hospitalized for 512 days with other inpatients carrying CRE with pKPI-6, and the isolate showed ∼83% similarity with a cluster of K. pneumoniae isolates carrying pKPI-6 in the XbaI-PFGE phylogeny (Fig. 1B). In addition, WGS of the plasmids revealed that a 714-bp region bracketed by 32-bp homologous regions was the only difference between pE105_IMP1 and pE013_IMP6 (Fig. 5A). This very small fragment appeared to have been removed by homologous recombination in pE105_IMP1 (Fig. 5B). Our results suggest that *bla*_IMP-6_ had disseminated via the transmission of pKPI-6, and spontaneous mutation may have generated the *bla*_IMP-1_-encoding plasmid providing broader antimicrobial resistance, resulting in increased fitness in the clinical setting.

This multi-institutional surveillance study uncovered the clonal dissemination of a plasmid encoding a specific carbapenemase IMP-6 and demonstrated that a seemingly clonal horizontal dissemination of CRE isolates had embraced heterogeneous minor subpopulations, which exhibited broadened antimicrobial resistance, stable carriage of *bla*_IMP-6_ through chromosomal integration, or heteroresistance related to covert *bla*_IMP_ transmission. Such diverse gene adaptations might also be common among CRE isolates carrying other carbapenemase genes. By multifaceted analysis of the plasmidome, this study revealed the vast regional dissemination of a carbapenemase-encoding plasmid, along with the presence of diverse derivatives that would ensure and facilitate the dissemination of carbapenemase genes in various environments, resulting in serious complications in clinical settings.

## MATERIALS AND METHODS

### CRE isolates and PFGE phylogenetic analysis.

We performed a CRE surveillance study of 1,507 patients hospitalized in 43 hospitals located in northern Osaka between December 2015 and January 2016 (10). In the present study, we analyzed 230 CRE isolates carrying *bla*_IMP_ obtained in the surveillance study, including 135 E. coli isolates and 95 K. pneumoniae isolates. All isolates were subjected to XbaI-digested PFGE for phylogenetic analysis (35). Dendrograms were generated from PFGE patterns by the UPGMA method using BioNumerics software (version 6.6; Applied Maths NV, Sint-Martens-Latem, Belgium).

### Classification of *bla*_IMP_ carriage by PFGE and Southern blotting.

The size and replicon type of *bla*_IMP_-harboring plasmids were determined by S1-nuclease-digested PFGE followed by Southern hybridization (S1 nuclease was obtained from TaKaRa Bio, Shiga, Japan). S1-PFGE and Southern blot hybridization for the *bla*_IMP-6_ and *repA* genes encoded on the IncN-type plasmid were performed as described in our previous study (12). The sizes of *bla*_IMP_-encoding plasmids were determined using BioNumerics software (version 7.5; Applied Maths NV). The modes of *bla*_IMP_ carriage were classified into seven groups based on the sizes and replicon types of the plasmids carrying *bla*_IMP_. The groups and their associated characteristics are as follows: group pKPI-6, a pKPI-6-like *bla*_IMP-6_-encoding plasmid (∼50 kbp, encoding *repA* for IncN plasmid); group IncN, a *bla*_IMP-6_-encoding plasmid (not ∼50 kbp, encoding *repA* for IncN plasmid); group non-IncN KP, a *bla*_IMP-6_-encoding plasmid (without *repA* for IncN plasmid) harbored by K. pneumoniae isolates; group IncF, a *bla*_IMP-6_-encoding plasmid (without *repA* for IncN plasmid) harbored by E. coli isolates; group double *bla*_IMP-6_, multiple plasmids with *bla*_IMP-6_ harbored by a single isolate; group chromosome, chromosomal *bla*_IMP-6_; group non-typeable, a *bla*_IMP-6_-encoding plasmid of unknown size; group IMP1, a *bla*_IMP-1_-carrier plasmid.

Isolates classified as chromosomal *bla*_IMP_ carriers were further analyzed to identify the location of *bla*_IMP_. In brief, I-CeuI endonuclease-digested PFGE followed by Southern blotting using probes for *bla*_IMP-6_ and 16S rRNA genes was performed to confirm the location of the *bla*_IMP_ gene in three E. coli isolates—E138, E300, and E302—as previously described (29).

### Antimicrobial susceptibility testing.

Susceptibility to ampicillin, ampicillin/sulbactam, piperacillin-tazobactam, piperacillin, cefotaxime, cefepime, imipenem, and meropenem was determined by the broth microdilution method according to the Clinical and Laboratory Standards Institute document M100-S28 (36). MICs of meropenem were determined using Etest (bioMérieux, Marcy l’Etoile, France), following the manufacturer’s instructions. E. coli ATCC 25922 was used as a control strain.

### Whole-genome sequencing and genomic analysis.

Genomic DNA for long- and short-read sequencing was extracted by using a DNeasy PowerSoil kit (Qiagen, Hilden, Germany). Short-read sequencing was conducted on an Illumina HiSeq 3000 sequencer using the KAPA library preparation kit (Kapa Biosystems, Woburn, MA) or on an Illumina MiSeq sequencer using the KAPA HyperPlus Library Preparation kit (Kapa Biosystems). Long-read sequencing was conducted on a Nanopore GridION sequencer (Oxford Nanopore Technologies, Oxford, UK) using sn SQK-LSK109 1D ligation sequencing kit and sn EXP-NBD103 native barcoding kit. The reads were assembled and polished using Unicycler (37). In cases where the complete plasmid sequences could not be constructed, sequences were assembled with CANU (version 1.8) (38) or flye (39) and improved using Pilon (40) or Racon (41). The PlasmidFinder (42) and ResFinder (43) databases were used to identify antimicrobial resistance genes and plasmid replicon types, respectively. A detailed analysis of the insertion sequence was performed using ISfinder (44). The sequences were annotated with RASTtk (45), and the genomic structures were compared with EasyFig (46). Plasmids similar to those found in this study were identified using BLAST.

### Transformation and bacterial transconjugation assay.

Plasmids were prepared from overnight cultures of E. coli isolates E033, E066, E174, and E305 and K. pneumoniae isolates E187, E188, E196, E208, and E328, using a plasmid miniprep kit (Qiagen). Electrocompetent TOP10 E. coli cells (Invitrogen, Waltham, MA) were electroporated with the extracted plasmids using a Gene Pulser Xcell system (Bio-Rad, Hercules, CA). After incubation in S.O.C. medium (Invitrogen) for 2 h (6 h for isolate E305), transformants were selected on Luria-Bertani (LB) agar supplemented with 0.125 μg/ml meropenem (2 μg/ml cefotaxime for isolate E305).

Bacterial conjugation assays were performed using the transformants as donors and the sodium azide-resistant E. coli strain TUM3456 (47) as a recipient. After mixing overnight cultures of donors and recipients at a 1:10 volumetric ratio, the mixture (10 μl) was incubated on LB agar for 24 h at 37°C. Transconjugants were selected on LB agar containing cefotaxime (2 μg/ml) and sodium azide (150 μg/ml). The conjugation frequency was calculated from the CFU as the number of transconjugants divided by the number of donors plus transconjugants.

### Determination of the plasmid copy number per host bacterial cell.

DNA of E. coli isolates E305 and E318, and E. coli transformants with plasmids pE188_IMP6 and pE305_IMP6_single_ (T188 and T305, respectively) was extracted using the DNA minikit (Qiagen). Using qPCR, the copy numbers of the *repA2* gene on plasmids pE305_IMP6 and pE318_IMP6 and the *bla*_IMP-6_ gene on pE188_IMP6 were compared to the copy number of the *rrsA* gene encoding 16S rRNA on the chromosome. qPCRs were carried out using Thunderbird SYBR qPCR Mix (Toyobo Life Science, Osaka, Japan) on a LightCycler 96 system (Roche Life Science, Penzberg, Germany). Primers used for this assay are list in Table S4 in the supplemental material. qPCR analysis was performed using data from repeated experiments (*n* = 6), and the plasmid copy number per cell was calculated from cycle threshold (*C_T_*) values using the comparative *C_T_* method (48).

10.1128/mSystems.00759-20.9 TABLE S4 Primers used in this study. Download Table S4, PDF file, 0.3 MB. Copyright © 2020 Abe et al. 2020 Abe et al. This content is distributed under the terms of the Creative Commons Attribution 4.0 International license.

### Determination of the copy number of *bla*_IMP-6_ per plasmid.

Plasmids of E. coli isolates E305 and E318 were extracted using a plasmid miniprep kit (Qiagen). Using qPCR, the copy numbers of the *bla*_IMP-6_ gene were compared to those of the *repA2* gene on plasmids pE305_IMP6 and pE318_IMP6. qPCRs were carried out using Thunderbird SYBR qPCR Mix on a LightCycler 96 System. Primers used for this assay are listed in Table S4. qPCR analysis was performed using data from repeated experiments (*n* = 5), and the *bla*_IMP-6_ copy number per plasmid was calculated from *C_T_* values using the comparative *C_T_* method.

### Transcription of *bla*_IMP-6_.

E. coli isolates E305 and E318, and E. coli transformants T188 and T305 were incubated in LB broth until the optical density at 600 nm reached 0.3 to 0.4. The total RNA was extracted using the RNeasy minikit (Qiagen). RNA was treated with ReverTra Ace qPCR RT Master Mix with gDNA remover (Toyobo Life Science) to remove contaminating DNA and to reverse transcribe the RNA into cDNA. For quality control, DNase-treated RNA that had not been reverse transcribed was subjected to a DNA contamination test by qPCR. The *rrsA* gene encoding 16S rRNA served as an endogenous control for normalization. qPCRs were carried out using Thunderbird SYBR qPCR Mix on a LightCycler 96 system. Primers used for this assay are listed in Table S4. qPCR analysis was performed using data from repeated experiments (*n* = 7), and transcript levels were calculated from *C_T_* values using the comparative *C_T_* method.

### Data availability.

The WGS data are available from the DDBJ (DNA Data Bank of Japan) database under accession numbers AB616660, AP019402, AP019405, and AP022349 to AP022369. Raw data of isolate E305 are available at NCBI under accession numbers DRX184368 and DRX182679.
