# Supplementary material for: Characterization of the Plasmidome Encoding Carbapenemase and Mechanisms for Dissemination of Carbapenem-Resistant Enterobacteriaceae
Source: mSystems. 2020 Nov 10;5(6):e00759-20. doi: 10.1128/mSystems.00759-20 (PMC7657596; doi:10.1128/mSystems.00759-20)
Supplement: TABLE S1 [file mSystems.00759-20-st001.pdf]

Table S1. Replicon types of *bla*$_{IMP-6}$-carrier plasmids from representative isolates in each *bla*$_{IMP}$ carriage group.

Species	Isolate	Group	Plasmid	Plasmid replicon type
K. pneumoniae	E013	pKPI-6	pE013_IMP6	IncN
	E196	IncN	pE196_IMP6	IncN. IncFIBk, FIIk
	E278	IncN	pE278_IMP6	IncFIBk, FIIk,
	E208	Non IncN KP	pE208_IMP6	IncR
	E328	Non IncN KP	pE328_IMP6	Unknown
	E105	IMP1	pE105_IMP1	IncN
E. coli	E033	IncN	pE033_IMP6	IncN, IncFII
	E034	IncN	pE034_IMP6	IncN, IncFII
	E109	IncN	pE109_IMP6	IncN
	E294	IncN	pE294_IMP6	IncN, IncFIA, FII
	E308	IncN	pE308_IMP6	IncN, IncFIA
	E317	IncN	pE317_IMP6	IncN, IncFIB
	E319	IncN	pE319_IMP6	IncFIA
	E301	IncF	pE301_IMP6	IncFIA
	E303	IncF	pE303_IMP6	IncFIA
	E305	IncF	pE305_IMP6	IncFIA
	E309	IncF	pE309_IMP6	IncFIA
	E310	IncF	pE310_IMP6	IncFIA
	E312	IncF	pE312_IMP6	IncFIA
	E318	IncF	pE318_IMP6	IncFIA
	E119	Double *bla*$_{IMP}$	pE119_5kIMP6	IncN
	E119	Double *bla*$_{IMP}$	pE119_6kIMP6	Unknown
