# Supplementary figures and images for: Characterization of the Plasmidome Encoding Carbapenemase and Mechanisms for Dissemination of Carbapenem-Resistant Enterobacteriaceae
Source: mSystems. 2020 Nov 10;5(6):e00759-20. doi: 10.1128/mSystems.00759-20 (PMC7657596; doi:10.1128/mSystems.00759-20)

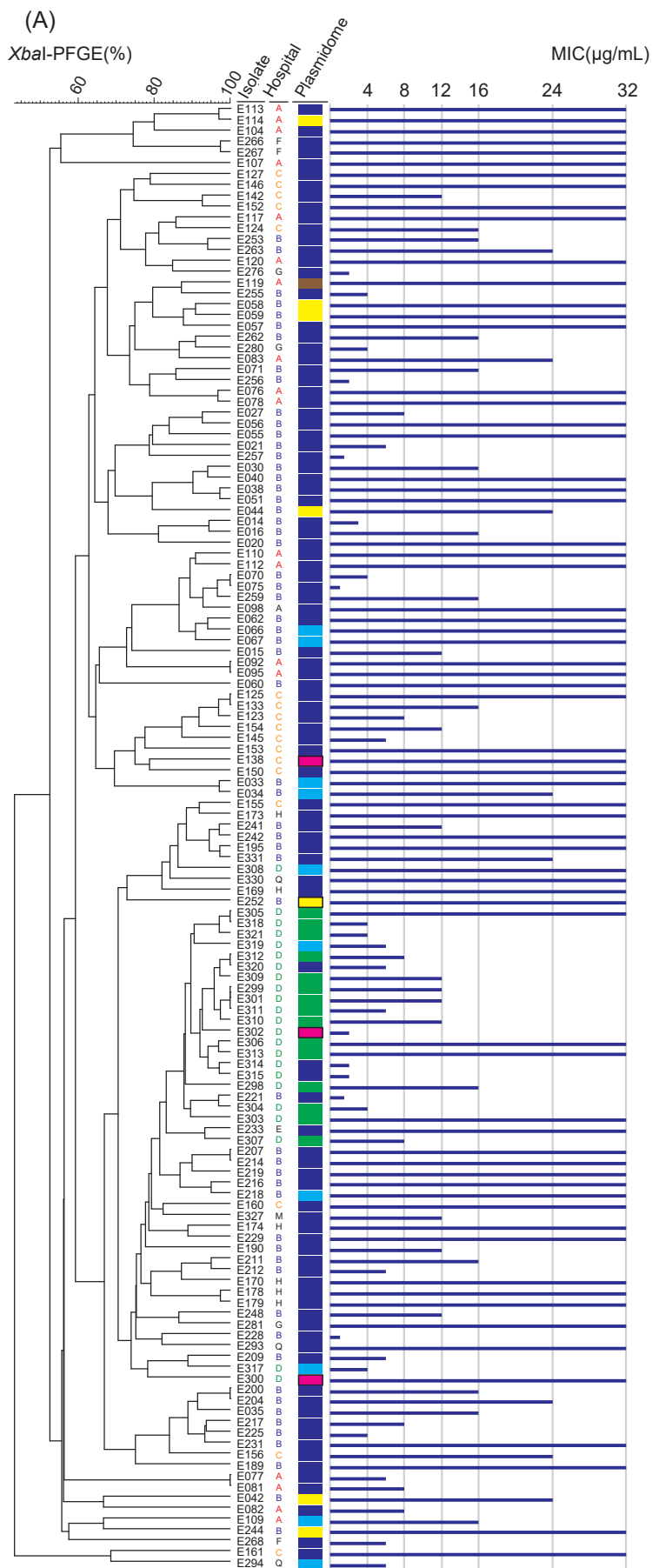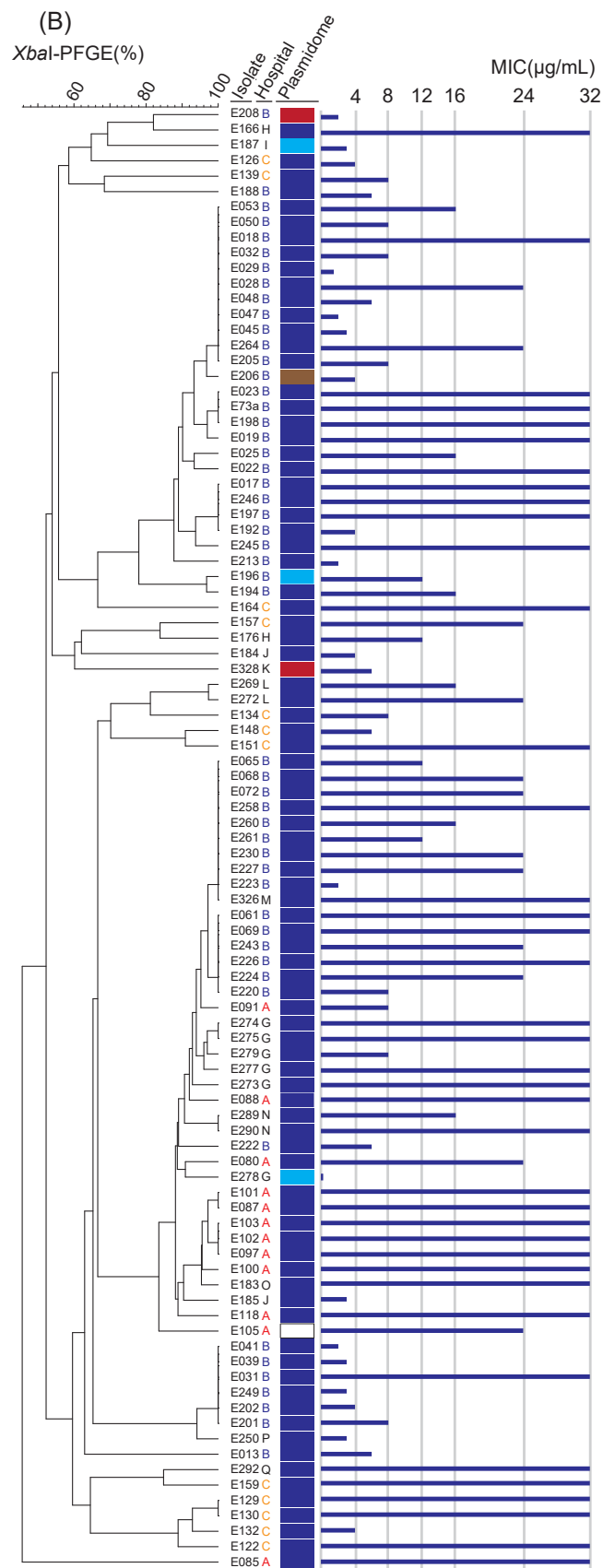

Supplement: FIG S4 [file mSystems.00759-20-sf004.pdf]
