# Supplementary material for: Characterization of the Plasmidome Encoding Carbapenemase and Mechanisms for Dissemination of Carbapenem-Resistant Enterobacteriaceae
Source: mSystems. 2020 Nov 10;5(6):e00759-20. doi: 10.1128/mSystems.00759-20 (PMC7657596; doi:10.1128/mSystems.00759-20)
Supplement: TABLE S2 [file mSystems.00759-20-st002.pdf]

Table S2A. Comparison of *ompC* and *ompF* harbored by *E. coli* isolates evaluated and an *E. coli* reference strain.

		ompC			***ompF***		
**Isolates**	**Group**	**Protain**	**Identity**	**Coverage**	**Protein**	**Identity**	**Coverage**
E038	pKPI-6	373	92%	100%	362	99%	100%
E070	pKPI-6	373	92%	100%	362	99%	100%
E083	pKPI-6	373	92%	100%	362	99%	100%
E110	pKPI-6	373	92%	100%	362	99%	100%
E146	pKPI-6	373	92%	100%	362	99%	100%
E160	pKPI-6	373	92%	100%	362	99%	100%
E173	pKPI-6	373	92%	100%	87	99%	24%, PSC
E174	pKPI-6	373	92%	100%	362	99%	100%
E195	pKPI-6	373	92%	100%	362	99%	100%
E229	pKPI-6	373	92%	100%	362	99%	100%
E233	pKPI-6	373	92%	100%	362	99%	100%
E262	pKPI-6	373	92%	100%	362	99%	100%
E280	pKPI-6	373	92%	100%	362	99%	100%
E319	pKPI-6	373	92%	100%	235	98%	64%, PSC
E033	IncN	373	92%	100%	362	99%	100%
E034	IncN	373	92%	100%	362	99%	100%
E066	IncN	373	92%	100%	362	99%	100%
E067	IncN	373	92%	100%	362	99%	100%
E109	IncN	363	96%	100%	362	99%	100%
E218	IncN	369	83%	100%	82	99%	22%, PSC
E294	IncN	364	90%	100%	357	93%	100%
E308	IncN	373	92%	100%	87	99%	24%, PSC
E317	IncN	373	92%	100%	362	99%	100%
E299	IncF	373	92%	100%	235	98%	64%, PSC
E301	IncF	373	92%	100%	235	98%	64%, PSC
E303	IncF	373	92%	100%	235	98%	64%, PSC
E304	IncF	373	92%	100%	235	98%	64%, PSC
E305	IncF	373	92%	100%	235	98%	64%, PSC
E306	IncF	373	92%	100%	235	98%	64%, PSC
E307	IncF	373	92%	100%	235	98%	64%, PSC
E309	IncF	373	92%	100%	235	98%	64%, PSC
E310	IncF	373	92%	100%	235	98%	64%, PSC
E312	IncF	373	92%	100%	235	98%	64%, PSC
E318	IncF	373	92%	100%	235	98%	64%, PSC
E119	Double *bla*$_{IMP}$	373	92%	100%	362	99%	100%
E138	Chromosome	373	92%	100%	362	99%	100%
E300	Chromosome	373	92%	100%	235	98%	64%, PSC
E302	Chromosome	373	92%	100%	235	98%	64%, PSC

Table S2B. Comparison of *ompK35* and *ompK36* harbored by the *K. pneumonia* isolates evaluated and a *K. pneumoniae* reference strain.

		ompK36			***ompK35***		
**Isolates**	**Group**	**Protein**	**Identity**	**Coverage**	**Protein**	**Identity**	**Coverage**
E013	pKPI-6	365	92%	100%	359	100%	100%
E031	pKPI-6	365	92%	100%	359	100%	100%
E039	pKPI-6	365	92%	100%	359	100%	100%
E045	pKPI-6	367	93%	100%	229	100%	75%
E053	pKPI-6	367	93%	100%	229	100%	75%
E065	pKPI-6	367	93%	100%	359	100%	100%
E085	pKPI-6	370	90%	100%	359	100%	100%
E087	pKPI-6	367	93%	100%	317	100%	88%
E088	pKPI-6	367	93%	100%	317	100%	88%
E101	pKPI-6	367	93%	100%	317	100%	88%
E126	pKPI-6	365	92%	100%	359	100%	100%
E129	pKPI-6	372	100%	100%	359	100%	100%
E130	pKPI-6	372	100%	100%	359	100%	100%
E132	pKPI-6	372	100%	100%	359	100%	100%
E134	pKPI-6	367	93%	100%	359	100%	100%
E139	pKPI-6	0	0%	0%	359	100%	100%
E148	pKPI-6	365	92%	100%	359	99%	100%
E159	pKPI-6	365	93%	100%	359	100%	100%
E183	pKPI-6	367	93%	100%	317	100%	88%
E184	pKPI-6	370	90%	100%	359	100%	100%
E185	pKPI-6	367	93%	100%	315	100%	87%
E188	pKPI-6	364	90%	100%	359	100%	100%
E194	pKPI-6	367	93%	100%	229	100%	75%
E202	pKPI-6	365	92%	100%	359	100%	100%
E250	pKPI-6	365	92%	100%	359	100%	100%
E269	pKPI-6	370	90%	100%	359	100%	100%
E277	pKPI-6	367	93%	100%	359	100%	100%
E289	pKPI-6	367	93%	100%	359	100%	100%
E326	pKPI-6	367	93%	100%	359	100%	100%
E196	IncN	367	93%	100%	229	100%	75%
E278	IncN	367	93%	100%	359	100%	100%
E208	Non IncN KP	367	93%	100%	359	100%	100%
E328	Non IncN KP	372	100%	100%	359	100%	100%
E105	IMP1	367	93%	100%	88	88%	12%
