# Supplementary material for: Characterization of the Plasmidome Encoding Carbapenemase and Mechanisms for Dissemination of Carbapenem-Resistant Enterobacteriaceae
Source: mSystems. 2020 Nov 10;5(6):e00759-20. doi: 10.1128/mSystems.00759-20 (PMC7657596; doi:10.1128/mSystems.00759-20)
Supplement: TABLE S3 [file mSystems.00759-20-st003.pdf]

Table S3. The location of ESBL genes carried *bla*$_{IMP}$-carrier isolates.

Species	Isolate	Group	Plasmid carrying *bla*$_{IMP-6}$	ESBL genes on plasmid with *bla*$_{IMP-6}$	Other ESBL genes carried by the isolate
E. coli	E146	pKPI-6	pE146_IMP6	*bla*$_{CTX-M-2}$	(-)
	E033	IncN	pE033_IMP6	(-)	(-)
	E034	IncN	pE034_IMP6	(-)	(-)
	E109	IncN	pE109_IMP6	*bla*$_{CTX-M-2}$	(-)
	E294	IncN	pE294_IMP6	*bla*$_{CTX-M-2}$, *bla*$_{TEM-1B}$	*bla*$_{CTX-M-14}$
	E308	IncN	pE308_IMP6	(-)	bla$_{CTX-M-15}$, *bla*$_{OXA-1}$
	E317	IncN	pE317_IMP6	*bla*$_{CTX-M-2}$	*bla*$_{TEM-1B}$
	E319	IncN	pE319_IMP6	(-)	(-)
	E298	IncF	pE298_IMP6	(-)	(-)
	E299	IncF	pE299_IMP6	(-)	(-)
	E301	IncF	pE301_IMP6	(-)	(-)
	E303	IncF	pE303_IMP6	(-)	(-)
	E304	IncF	pE304_IMP6	(-)	(-)
	E305	IncF	pE305_IMP6	(-)	(-)
	E306	IncF	pE306_IMP6	(-)	(-)
	E307	IncF	pE307_IMP6	(-)	(-)
	E309	IncF	pE309_IMP6	(-)	*bla*$_{CTX-M-2}$
	E310	IncF	pE310_IMP6	(-)	(-)
	E311	IncF	pE311_IMP6	(-)	(-)
	E312	IncF	pE312_IMP6	(-)	(-)
	E313	IncF	pE313_IMP6	(-)	(-)
	E318	IncF	pE318_IMP6	(-)	*bla*$_{CTX-M-14}$, *bla*$_{TEM-1B}$
	E321	IncF	pE321_IMP6	(-)	(-)
	E119	Double *bla*$_{IMP}$	pE119_5kIMP6	*bla*$_{CTX-M-2}$	(-)
	E119	Double *bla*$_{IMP}$	pE119_6kIMP6	(-)	(-)
K. pneumoniae	E013	pKPI-6	pE013_IMP6	*bla*$_{CTX-M-2}$	(-)
	E126	pKPI-6	pE126_IMP6	*bla*$_{CTX-M-2}$	*bla*$_{SHV-11}$
	E129	pKPI-6	pE129_IMP6	*bla*$_{CTX-M-2}$	(-)
	E130	pKPI-6	pE130_IMP6	*bla*$_{CTX-M-2}$	(-)
	E132	pKPI-6	pE132_IMP6	*bla*$_{CTX-M-2}$	*bla*$_{SHV-38}$
	E134	pKPI-6	pE134_IMP6	*bla*$_{CTX-M-2}$	*bla*$_{SHV-11}$
	E148	pKPI-6	pE148_IMP6	*bla*$_{CTX-M-2}$	*bla*$_{SHV-60}$
	E188	pKPI-6	pE188_IMP6	*bla*$_{CTX-M-2}$	(-)
	E196	IncN	pE196_IMP6	*bla*$_{CTX-M-2}$	*bla*$_{SHV-1}$
	E278	IncN	pE278_IMP6	*bla*$_{CTX-M-2}$	*bla*$_{SHV-11}$
	E208	Non IncN KP	pE208_IMP6	*bla*$_{CTX-M-15}$	*bla*$_{DHA-1}$, *bla*$_{SHV-12}$, *bla*$_{SHV-28}$
	E328	Non IncN KP	pE328_IMP6	(-)	*bla*$_{SHV-11}$
	E105	IMP1	pE105_IMP1	*bla*$_{CTX-M-2}$	(-)
