# Supplementary material for: Characterization of the Plasmidome Encoding Carbapenemase and Mechanisms for Dissemination of Carbapenem-Resistant Enterobacteriaceae
Source: mSystems. 2020 Nov 10;5(6):e00759-20. doi: 10.1128/mSystems.00759-20 (PMC7657596; doi:10.1128/mSystems.00759-20)
Supplement: TABLE S4 [file mSystems.00759-20-st004.pdf]

Table S4. Primers used in this study.

Primer name	Primer sequence (5'' to 3'')	Usage
**Southern hybridization of *bla*$_{IMP}$ and *repA* for IncN plasmid**		
IMPF	GCTACCGCAGCAGAGTCTTT	Probing for *bla*$_{IMP}$
IMPR	CAAGAGTGATGCGTCTCCAA	
repAF	AAGCAGTCTAACGAGCTTACCG	Probing for *repA* for IncN plasmid
repAR	GGAAATTCGCCCATCTTCAT	
**qPCR for transcription of *bla*$_{IMP-6}$**		
IMP6-qPCRF	GGTTTAGGCAATTTGGGTGA	*bla*$_{IMP-6}$ transcription
IMP6-qPCRR	CAAGAGTGATGCGTCTCCAA	
rrsA-F	GTGGCTACGATTGCATTCCA	Internal control with *rrsA* on chromosome
rrsA-R	TAGCGCATTAATTACGCCAA	
**qPCR for plasmid copy numbers of pE188_IMP6 and pE305_IMP6$_{single}$**		
IMP6-qPCRF	GGTTTAGGCAATTTGGGTGA	Plasmid copy number for pE188_IMP6
IMP6-qPCRR	CAAGAGTGATGCGTCTCCAA	
305F	AACAGCGGCCATTTGTTT	Plasmid copy number for pE305_IMP6
305R	GTCGCAGACAGAAAATGCAG	
rrsA-F	GTGGCTACGATTGCATTCCA	*rrsA* on chromosome
rrsA-R	TAGCGCATTAATTACGCCAA	
**qPCR for *bla*$_{IMP-6}$ copy numbers on plasmids pE305_IMP6, pE305_IMP6$_{single}$ and pE318_IMP6**		
IMP6-qPCRF	GGTTTAGGCAATTTGGGTGA	*bla*$_{IMP-6}$ copy number
IMP6-qPCRR	CAAGAGTGATGCGTCTCCAA	
305F	AACAGCGGCCATTTGTTT	*repA2* on plasmid
305R	GTCGCAGACAGAAAATGCAG	
**qPCR for plasmid copy numbers of pE305_IMP6 and pE318_IMP6**		
305F	AACAGCGGCCATTTGTTT	*repA2* copy number
305R	GTCGCAGACAGAAAATGCAG	
rrsA-F	GTGGCTACGATTGCATTCCA	*rrsA* on chromosome
rrsA-R	TAGCGCATTAATTACGCCAA	
